# Cervical Chylous Leakage Combined With Chylothorax: A Case Report of a Rare Complication Postretroperitoneal Surgery and Its Management: A Case Report of Chylous Leakage

**DOI:** 10.1155/2024/8820322

**Published:** 2024-10-22

**Authors:** Yao Zhou, Mingde Ding, Qiang Shi, Jing Wang, Guanghai Liu, Qianqian Zhang

**Affiliations:** ^1^Department of Obstetrics and Gynecology, The Second Affiliated Hospital of Shandong First Medical University, Tai'an 271000, China; ^2^Department of Medical Imaging, The Second Affiliated Hospital of Shandong First Medical University, Tai'an 271000, China; ^3^Doppler Ultrasonic Department, The Second Affiliated Hospital of Shandong First Medical University, Tai'an 271000, China; ^4^Department of Obstetrics and Gynecology, Central Hospital Affiliated to Shandong First Medical University, Jinan 250000, China

**Keywords:** cervical chylous leakage, chylothorax, conservative treatment, postretroperitoneal surgery

## Abstract

Chylous leakage is an uncommon and serious clinical condition, especially occurring after retroperitoneal operations. Here, we report a case of cervical chylous leakage combined with chylothorax in a 57-year-old woman postretroperitoneal surgery, and our conservative approach led to resolution/clinical improvement. The causes of this rare complication are discussed. We considered that the venous thrombosis, the increased intra-abdominal pressure, or the patient positioning during the surgery may lead to the chylous particles leakage and chylothorax. Because of its rarity, we hope this case report will improve clinicians' understanding of cervical chylous leakage combined with chylothorax and provide suitable treatment options for future clinical reference.

## 1. Introduction

Chylous leakage is defined as a pathologic accumulation of chyle in the neck, thoracic, or peritoneal cavity. It is caused by unrecognized disruption of major lymphatic channels, primarily occurring after operations or other nontraumatic reasons [1]. Cervical chylous leakage or chylothorax after abdominal surgery is rare. Over a 5-year period, this is the first case of chylous leakage after abdominal surgery reported in our Obstetrics and Gynecology surgical unit. A chyle leak may lead to malnutrition, compromise of the immune system, electrolyte abnormalities, and an increased risk of infection. Treatment is always challenging and may be conservative, based on diet or parenteral nutrition, or surgical intervention [2]. Herein, we describe a rare case of cervical chylous leakage combined with chylothorax after the transperitoneal laparoscopic operation, which was successfully managed with conservative treatment.

## 2. Case Description

A 57-year-old woman was referred to our hospital for the surgical management of Cystocele (III grade) and Uterine prolapse (I grade) based on the International Continence Society/International Urogynecology Association Pelvic Organ Prolapse Quantification (POP-Q) system. Preoperative physical examination and laboratory data revealed no remarkable findings. Ultrasound revealed multiple thyroid nodules, thus thyroid puncture and cell biopsy were performed, and no significant heteromorphic cells were found. Eight days after thyroid puncture, the patient underwent laparoscopic total hysterectomy and bilateral adnexectomy (Pan-hysterectomy), Uterosacral ligament suspension, Anterior vaginal wall repair (Colporrhaphy), and Marshall–Marchetti–Krantz urethropexy.

**Figure 1:**
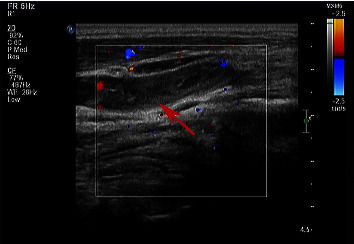
Ultrasonography of the cervical mass showed the left external jugular vein is widened which appears to be filled with no echo in its main trunk.

**Figure 2:**
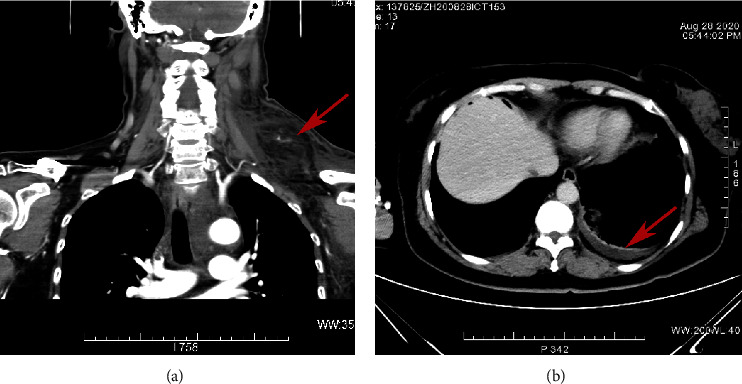
A computed tomography scan reveals suspicious neck cellulitis (a) and left pleural effusion (b).

On the first day after surgery, the patient developed neck discomfort with restriction movement to left. Physical examination revealed a new soft, fluctuating mass in her left lateral neck, sized about 9 × 8 cm. The Color Duplex Ultrasonography of the cervical mass showed the left external jugular vein is widened and extends downward to the subclavian vein inlet area, which appears to be a thrombus (Figure 1). Patient subsequently developed dyspnea gradually and following CT on Day 2 postsurgery revealed suspicious neck cellulitis and bilateral pleural effusion (Figure 2). Blood analysis showed that the percentage of neutrophils was 81.4% (Reference range 50%–70%) and the percentage of lymphocytes was 12.1 (Reference range 20–40). Coagulation analysis showed that D dimer was 1.85 mg/L (Reference range 0–0.55), procalcitonin was 0.114 ng/mL (Reference range 0–0.05), inorganic ions showed potassium 3.18 mmol/L (Reference range 3.5–5.3), calcium was 2.03 mmol/L (Reference range 2.11–2.52), liver function showed albumin was 25.6 g/L (Reference range 40–55), and total protein was 47.8 g/L (Reference range 65–85). To further clarify the nature of the effusion, 3 mL pleural effusion was extracted, and the pleural fluid analysis revealed 5.03 mmol/L of triglycerides with lymphocytes predominant. Chylous leakage was thus highly suspected.

From the second to the 10th-day postsurgery, the patient was managed conservatively. Total parenteral nutrition (TPN) and external application of mirabilite was our treatment of choice for this complication. On the eighth-day postsurgery, repeated CT revealed the amount of bilateral pleural effusion did significantly decrease. At the same time, the symptom of dyspnea was significantly abated. These therapeutic approaches proved to be effective. For symptomatic management, the patient received, as needed, prophylactic antibiotic therapy which consisted of intravenous cephalosporin. In addition, intravenous Ambroxol and inhaled budesonide were given for her continuous cough and expectoration.

Moreover, the patient was followed up for 2 years after the operation. During the follow-up survey, she was generally in good condition with no other discomfort or new symptoms.

## 3. Discussion

Chylous leakage results from the accumulation of lymphatic fluid in the pleural space due to an obstruction or leakage of the thoracic duct or one of its contributors. Although rare, chylous leakage is a serious, even life-threatening event if not properly treated.

Chylous leakage is a rare and serious complication. Diagnosis is based on the triglyceride and cholesterol content of pleural fluid or abdominal cavity effusion obtained with thoracentesis. Staatset al [3]. have suggested that a pleural fluid with a triglyceride level of > 110 mg/dL (1.24 mmol/L) can support the diagnosis of Chylous leakage and give this test a high specificity. In our case, the initial presentation of neck discomfort with soft tissue swelling made it difficult to be recognized. However, the analysis of the pleural effusion fluid with 5.03 mmol/L of triglycerides supported the diagnosis of chylous leakage. This provided a new clinical basis for early diagnosis of Chylous leakage.

The thoracic duct originates as the cisterna chyli on the anterior surface of the bodies of the first and second lumbar vertebrae and enters the venous system at the subclavian-internal jugular vein junction on the left [4]. The function of the thoracic duct is to transport chyle back to the bloodstream. The thoracic duct transports 1.5–4 L of chyle in a normal adult per day [5]. Most of this fluid is from the intestinal lymphatics and liver, which transport long-chain fatty acids and protein absorbed through intestinal lacteals [6].

The main risk factors for postesophagectomy chylothorax include the anatomical variations of the thoracic duct (TD), neoadjuvant therapies, and squamous histology [7]. High BMI is a protective factor, BMI < 25 was a risk factor [8, 9]. Patients' age, sex, BMI, and serum albumin level have no significant correlation with the risk for developing postsurgery chylothorax [10]. This patient was a 57-year-old middle-aged female with no prior cerebrovascular disease. Her BMI was in the normal range (22.2 kg/m^2^) and her preoperative serum albumin level was also in the normal range (46.6 g/L, Reference range 40–55 g/L). No clear risk factors were present.

The main cause of chylothorax is traumatic, typically postsurgical. Causes of nontraumatic chylous leakage include a wide range of differential diagnoses, such as Malignant, Lymphatic disorders, Sarcoidosis, Infections, and Venous thrombosis [11]. In this case, the possible mechanisms of the lymphatic leakage are as follows. First, thrombogenesis. We suspect the thrombosis in the left external jugular-subclavian vein junction may block the lymphatic reflux, causing sudden increase of intraductal pressure and initiating a chylous leak. Second, we suspect that the CO_2_ pneumoperitoneum has increased the intra-abdominal pressure, and thus the compression on the cisterna chili increase the amount of chyle, resulting in increased permeability or rupture of the lymphatic wall, subsequently the leakage of chylous fluid into the neck and thoracic cavity. Third, the longtime head-down position during laparoscopic surgery may increase the amount of chyle, resulting in increased hydrostatic pressure. In addition, the patient had undergone a thyroid puncture. Some lymphatics wall of the lymph branches may have been infiltrated or damaged during the procedure, which may lead to spontaneous lymphatic leakage.

In order to reduce the risk of thrombosis, preoperative venous thromboembolism (VTE) evaluation should be routinely performed for all patients before surgery. The Caprini RAM is comprehensive method which includes 39 individual risk factors such as age, weight, personal history of VTE and also surgery-related variables [12]. Patients are divided into low-risk group, medium-risk group and high-risk group according to their scores. The low-risk groups only need basic prevention, such as encouraging early ambulation after surgery. The medium-risk groups need additional physical prevention such as graduated compression stockings and intermittent pneumatic compression devices. And patients of high-risk group need further drug treatment, such as mini-dose unfractionated heparin. In addition, preventive measures should also be taken according to the result to reduce the incidence of thrombosis, intra-abdominal pressure should not be too high during the operation.

As the lymphatic system plays an essential role in fat absorption and immune response, a lymphatic leak may cause life-threatening malnutrition and immunodeficiency [13]. The rapid accumulation of chyle in a confined space in the neck can lead to swelling which can compromise respiration if not identified and drained promptly. Slow leaks can lead to the formation of chylous lymphoceles, or “chylomas,” weeks or months later. Prolonged chylous leakage can lead to nutritional deficiency, hypovolemia immunosuppression, and hemorrhagic complications [14]. Therefore, making the right treatment decision is as important as the diagnosis. In the previous studies, if the lymphatic drainage is lower than 500 mL/day, conservative treatment may be preferred, such as prophylactic antibiotics and nutritional modification. On the contrary, in cases of a higher volume of chyle drainage (over 500 mL/day), continual flow, and nutritional or immunological insufficiency, surgical treatment may be more effective [15].

In this case, the patient was managed successfully with conservative approaches which include bed rest, external application and total parenteral nutrition. It is an effective method for the management of chylous leakage, at least in the case of the lower volume of chyle drainage. The therapeutic rationale for TPN is to minimize intestinal lymphatic transport of chylomicrons, allowing the areas of lymphatic leakage to seal. However, due to the rarity retroperitoneal surgery, we cannot offer any recommendation on when to discontinue conservative management and proceed with surgery.

In conclusion, early identification of chylous leakage is very important, and it should be treated promptly once it is identified. In addition, the suitable candidates for early intervention have good performance status with conservative management.

## Data Availability

All data generated or analyzed during this study are included within the article and are also available from the corresponding author upon request.
